# Early pregnancy fasting plasma glucose and risk factors: impact on gestational diabetes mellitus and other adverse perinatal outcomes in singleton and twin pregnancies

**DOI:** 10.3389/fmed.2026.1851313

**Published:** 2026-08-03

**Authors:** Ran Wang, Ying Liu

**Affiliations:** 1 Department of Obstetrics and Gynecology, Ningjin County Hospital, Xingtai, Hebei, China; 2 Department of Central Laboratory, The Third Affiliated Hospital of Jinzhou Medical University, Jinzhou, Liaoning, China

**Keywords:** early pregnancy fasting plasma glucose, gestational diabetes mellitus, perinatal outcomes, risk factors, singleton pregnancy, twin pregnancy

## Abstract

**Background:**

Gestational diabetes mellitus (GDM) poses distinct challenges across different pregnancy types, yet whether the roles of early metabolic markers and conventional risk factors vary between singleton and twin pregnancies remains unclear. This study sought to clarify the associations of early pregnancy fasting plasma glucose (FPG) and established risk factors with GDM and adverse perinatal outcomes in both pregnancy settings, with a particular focus on heterogeneity across the two groups.

**Methods:**

This retrospective study enrolled 5,532 singleton and 175 twin pregnancies from Ningjin County Hospital, Hebei Province, between January 1, 2019, and December 31, 2024. Data collected encompassed maternal baseline characteristics, early pregnancy FPG, GDM diagnosis (based on IADPSG criteria), and perinatal outcomes. Analytical approaches comprised multivariate logistic regression, interaction analysis, SHAP (Shapley additive explanations) analysis, and receiver operating characteristic (ROC) curve evaluation.

**Results:**

GDM incidence was significantly higher in twin than singleton pregnancies (30.29% vs. 21.98%, *p* < 0.05), as were adverse perinatal outcomes (preeclampsia, preterm birth, cesarean section; all *p* < 0.05). In singletons, early pregnancy FPG and all traditional risk factors correlated with GDM (*p* < 0.05); in twins, GDM history and PCOS associations with GDM were attenuated (not significant). Interaction analysis showed pre-pregnancy BMI and PCOS had stronger GDM predictive effects in singletons (*p*-interaction < 0.05). SHAP analysis confirmed lower contributions of GDM history and PCOS in twins, while early pregnancy FPG was the top predictor in both. Individual predictors had limited GDM predictive value (AUCs < 0.6), but combining early pregnancy FPG with all risk factors improved performance (AUCs: 0.783 for singletons, 0.784 for twins). Early pregnancy FPG and risk factors correlated with adverse outcomes, with more pronounced effects in twins.

**Conclusion:**

Twin pregnancy is a high-risk condition for GDM and adverse outcomes. Pre-pregnancy BMI and PCOS are more strongly predictive of GDM in singleton pregnancies, while early pregnancy FPG serves as a universal predictor in both types. Individualized management strategies based on pregnancy type are warranted, with enhanced monitoring for twin pregnancies.

## Introduction

Gestational diabetes mellitus (GDM), a common metabolic complication unique to pregnancy, is pathogenically linked to insulin resistance and hormonal disruptions triggered by the gestational state (1). Beyond elevating the likelihood of maternal morbidities such as preeclampsia and delivery via cesarean section, GDM exerts adverse perinatal effects, including fetal macrosomia and neonatal hypoxia, thereby imposing substantial risks on the short- and long-term health trajectories of both mothers and infants (2, 3). Concurrently, the expanding utilization of assisted reproductive technologies has contributed to a persistent upward trend in the prevalence of twin gestations (4). Given their amplified metabolic demands and more complex physiological adaptations, twin pregnancies are universally recognized as a high-risk cohort for developing GDM and experiencing poor pregnancy outcomes (5). Despite this, there remains a paucity of systematic comparative investigations exploring the divergent etiological mechanisms of GDM and the differential impacts of risk factors between singleton and twin pregnancies.

Fasting plasma glucose (FPG) in early pregnancy serves as a key indicator for assessing maternal glucose metabolism and has been confirmed to be closely related to the risk of developing GDM in the second trimester (6). Furthermore, traditional risk factors such as advanced maternal age, pre-pregnancy overweight/obesity, and a family history of diabetes are well-established independent predictors of GDM (7). However, the majority of existing epidemiological studies have centered on singleton pregnancies. Consequently, empirical evidence regarding the efficacy of combining early pregnancy FPG with traditional risk factors for predicting GDM in twin pregnancies, as well as the contrasting effects of these factors on adverse perinatal events across the two pregnancy types, remains inadequate (8). This knowledge gap hinders the development of individualized, evidence-based strategies for early GDM screening and intervention tailored to different pregnancy types.

Therefore, this study enrolled singleton and twin pregnant women managed at Ningjin County Hospital, Hebei Province, to systematically investigate the associations of early pregnancy FPG and various risk factors with the incidence of GDM and adverse perinatal outcomes. A core objective was to elucidate the potential heterogeneity in these associations between singleton and twin pregnancies. The findings derived from this study are expected to delineate the distinct risk profiles and key predictive biomarkers for GDM in different gestational settings. Ultimately, this work aims to provide a scientific foundation for refining antenatal care guidelines and mitigating the incidence of GDM and its associated adverse pregnancy complications.

## Methods

### Study population

**Figure 1:**
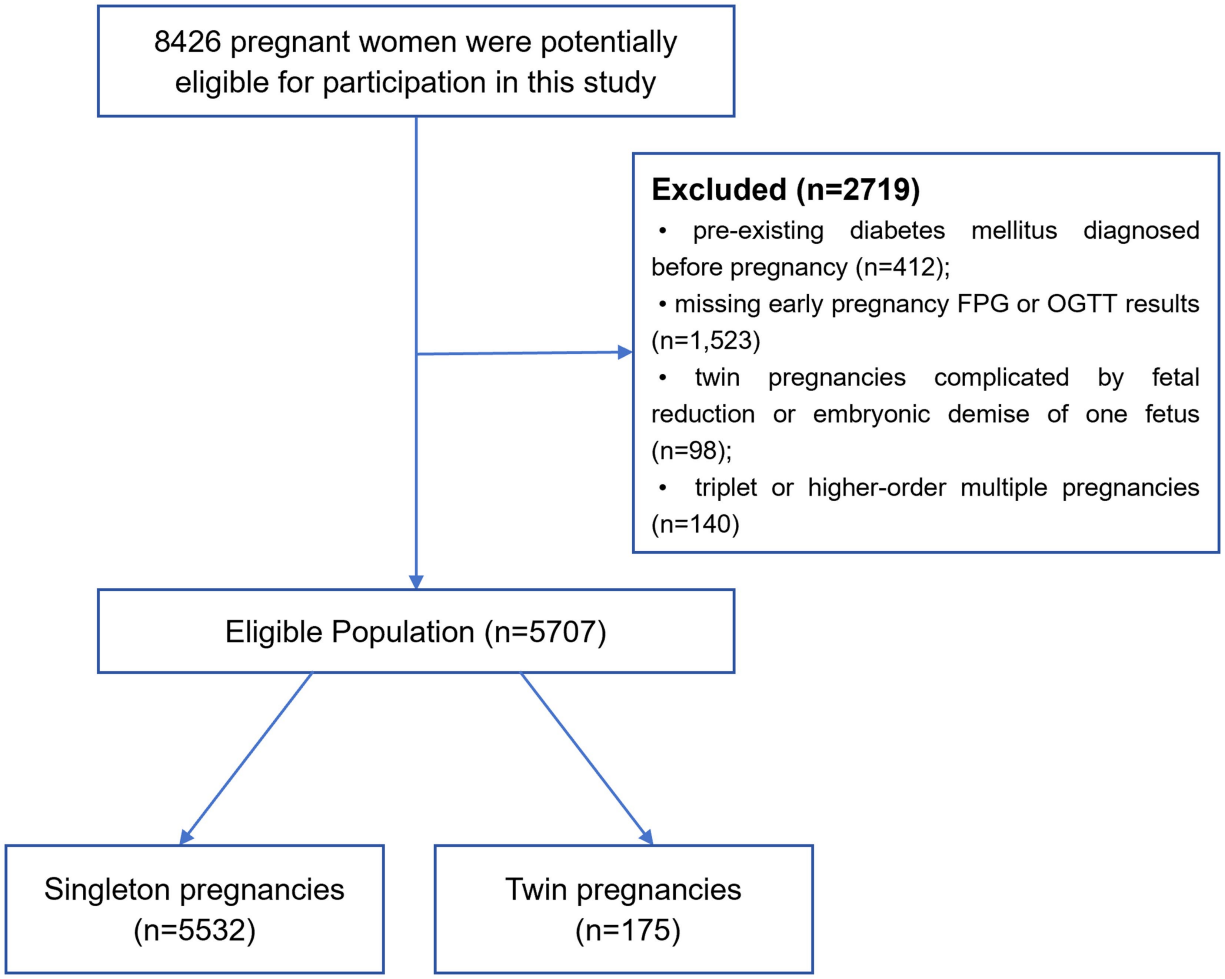
CONSORT flow chart for screening of study subjects.

During the study period (January 1, 2019 to December 31, 2024), a total of 8,426 pregnant women received regular antenatal care and delivered at Ningjin County Hospital. Among them, 2,719 were excluded from the analysis for the following reasons: pre-existing diabetes mellitus diagnosed before pregnancy (*n* = 412); missing early pregnancy FPG or oral glucose tolerance test (OGTT) results (*n* = 1,523); incomplete perinatal outcome records (*n* = 546); twin pregnancies complicated by fetal reduction or embryonic demise of one fetus (*n* = 98); and triplet or higher-order multiple pregnancies (*n* = 140). After exclusions, a total of 5,707 pregnant women were ultimately included in this study, comprising 5,532 singleton pregnancies and 175 twin pregnancies. A CONSORT-style flow diagram of participant selection is presented in Figure 1.

The inclusion criteria were as follows: age ≥ 18 years; confirmed singleton or twin pregnancy; absence of mental disorders or cognitive impairments, with the ability to cooperate with routine antenatal examinations and related tests; complete clinical medical records permitting extraction of all required study data. The exclusion criteria were as follows: pre-existing diabetes mellitus diagnosed before pregnancy (i.e., diabetes complicating pregnancy); twin pregnancies complicated by complex conditions, or those undergoing fetal reduction or experiencing embryonic demise of one fetus; stillbirth or intrauterine fetal demise during pregnancy; triplet or higher-order multiple pregnancies; missing medical records or incomplete key study indicators. To assess the potential for selection bias, we compared the baseline characteristics of the excluded population (*n* = 2,719) with those of the included population (*n* = 5,707). No statistically significant differences were observed for these key variables (all *p* > 0.05), suggesting that the missing data were likely missing at random (Table 1). This supports the internal validity of our findings.

**Table 1:** Comparison of baseline characteristics between excluded and included populations.

Indicators	Excluded population (*n* = 2,719)	Included population (*n* = 5,707)	*t*/*χ*^2^	*p*
Age			0.275	0.600
< 35 years	2,435 (89.55)	5,132 (89.92)		
≥ 35 years	284 (10.45)	575 (10.08)		
Pre-pregnancy BMI			0.205	0.651
< 25 kg/m^2^	2,271 (83.52)	4,872 (85.37)		
≥ 25 kg/m^2^	448 (16.48)	835 (14.63)		
FPG in early pregnancy, mmol/L	4.65 ± 0.42	4.66 ± 0.47	0.944	0.345
Pregnancy type [*n* (%)]			0.109	0.742
Single	2,632 (96.80)	5,532 (96.93)		
Twin	87 (3.20)	175 (3.07)		

Values are presented as number (%) or mean ± standard deviation.

BMI, body mass index; FPG, fasting plasma glucose.

### Diagnostic and definition criteria

#### Diagnostic criteria for GDM

GDM was identified in line with the one-step method proposed in the 2010 International Association of Diabetes and Pregnancy Study Groups (IADPSG) consensus criteria, as endorsed by the World Health Organization (WHO) in 2013 (9). All pregnant women who had not been previously diagnosed with diabetes were administered a 75-g OGTT during 24 to 28 weeks of gestation. GDM was confirmed if any of the following cutoff values were reached: FPG ≥ 5.1 mmol/L, 1-h plasma glucose ≥ 10.0 mmol/L, or 2-h plasma glucose ≥ 8.5 mmol/L.

#### Definition of high-risk population for GDM

Referring to previous research (10, 11) and the Chinese “Guideline for the Diagnosis and Management of Hyperglycemia in Pregnancy (2022) [Part I]” (12), pregnant women were categorized as high-risk for GDM if they satisfied any of the following conditions: age ≥ 35 years; pre-pregnancy body mass index (BMI) ≥ 25 kg/m^2^; a history of GDM in a prior pregnancy; a family history of diabetes; concurrent polycystic ovary syndrome (PCOS); a history of macrosomic delivery; a history of fetal malformations, and other related factors.

#### Definitions of secondary outcomes

(1) For singleton pregnancies, the diagnostic standards for large for gestational age (LGA) and small for gestational age (SGA) were based on the “Growth Evaluation Standards for Newborns at Different Gestational Ages” released by the National Health Commission of the People’s Republic of China in 2022. (2) For twin pregnancies, the diagnostic criteria for LGA and SGA were derived from the twin birth weight percentiles established by researchers from West China Second University Hospital, Sichuan University (13). These percentiles were obtained through the analysis of twin birth weight data of newborns delivered in China between October 2006 and September 2015. Newborns with a birth weight exceeding the 90th percentile (P90) for their gender and gestational age were defined as LGA, whereas those with a birth weight below the 10th percentile (P10) were classified as SGA.

### Data collection

This study extracted relevant data of all participants from the hospital’s electronic medical record system, covering three core categories: (1) Baseline demographic and clinical characteristics: age, gravidity, parity, pre-pregnancy BMI, mode of conception (spontaneous conception or assisted reproductive technology), previous GDM history, family history of diabetes, prior macrosomic infant delivery history, chronic hypertension coexisting with pregnancy, polycystic ovary syndrome (PCOS) history, and other high-risk pregnancy factors. (2) Laboratory measurement of FPG: Early pregnancy FPG was measured at the first prenatal visit. All participants were instructed to fast for at least 8 h overnight before blood sampling. Venous blood samples (3 mL) were collected in the morning (8:00–10:00 a.m.) using sodium fluoride tubes. Samples were centrifuged within 30 min of collection at 3,000 rpm for 10 min, and plasma glucose was measured using the glucose oxidase method on an automated biochemical analyzer (Beckman Coulter AU5800, Brea, CA, USA). All analyses were completed within 2 h of sample collection to ensure result stability. The 75-g OGTT for standard GDM diagnosis was performed strictly between 24 and 28 weeks of gestation for all women without pre-existing diabetes. (3) Perinatal adverse outcomes: preeclampsia, premature rupture of membranes, cesarean delivery, preterm birth, postpartum hemorrhage, macrosomia, neonatal hyperbilirubinemia, and neonatal asphyxia.

Standard diagnostic criteria were adopted for all perinatal outcome indicators, defined as follows: Preeclampsia was diagnosed as *de novo* hypertension (systolic blood pressure ≥ 140 mmHg and/or diastolic blood pressure ≥ 90 mmHg) occurring after 20 weeks of gestation, combined with proteinuria (≥ 300 mg per 24 h) or other maternal organ dysfunctions. Premature rupture of membranes was defined as fetal membrane rupture prior to the initiation of labor. Cesarean delivery referred to fetal delivery via surgical incisions on the maternal abdomen and uterus. Preterm birth was defined as delivery before 37 completed gestational weeks. Postpartum hemorrhage was defined as blood loss ≥ 500 mL within 24 h after vaginal delivery or ≥ 1,000 mL after cesarean delivery. Macrosomia was defined as neonatal birth weight ≥ 4,000 g irrespective of gestational age. Neonatal hyperbilirubinemia was defined as serum total bilirubin level exceeding the 95th percentile for the corresponding postnatal age in hours or meeting the threshold for phototherapy. Neonatal asphyxia was defined as 1-min Apgar score ≤ 7 or umbilical artery blood pH < 7.2, accompanied by clinical neurological symptoms.

### Statistical analysis

Statistical analysis was conducted utilizing SPSS version 26.0 (IBM Corp., Armonk, NY, USA). For continuous variables following normal distribution, descriptive statistics were presented as arithmetic mean accompanied by standard deviation (x̄ ± s), with between-group comparisons executed via the two-sample Student’s t-test. Continuous variables deviating from normality were summarized using median value alongside interquartile range [*M* (P25, P75)], and group differences were evaluated through the Mann–Whitney nonparametric procedure. Categorical data were displayed as absolute counts and relative frequencies (*n*, %), with intergroup comparisons carried out employing Pearson’s Chi-squared test or Fisher’s exact probability test when cell frequencies were insufficient.

In this study, early pregnancy FPG was analyzed as a continuous variable (mmol/L). To examine the relationships between first-trimester FPG, diverse predisposing factors, and the development of GDM as well as unfavorable perinatal events, multivariable logistic regression modeling was implemented, yielding adjusted odds ratios (aOR) with corresponding 95% confidence intervals (95% CI). Multiplicative interaction terms were introduced to explore potential effect modifications between gravidity type (singleton versus multiple gestation) and exposure variables on GDM incidence and perinatal morbidity.

Additionally, Shapley Additive exPlanations (SHAP) methodology was applied to quantify the relative importance of each predictor in contributing to GDM risk, generating visual representations of both the strength and polarity of variable effects. Discriminatory performance of first-trimester FPG, isolated risk indicators, and composite predictive algorithms was appraised through receiver operating characteristic (ROC) curve construction, with model discrimination quantified by the area under the ROC curve (AUC). All hypothesis tests were two-tailed, with statistical significance declared at *p* < 0.05.

### Ethics statement

This study was approved by the Ethics Committee of Ningjin County Hospital, Hebei Province (No. 2025046H).

## Results

### Baseline characteristics

A total of 5,707 pregnant women were enrolled (5,532 singleton, 175 twin pregnancies). Twin pregnancies had a significantly higher GDM incidence (30.29%) than singleton ones (21.98%, *p* < 0.05). In baseline characteristics, the twin group had higher proportions of women ≥ 35 years, gravidity ≥ 2, assisted reproductive technology users, PCOS patients and high-risk pregnancies, as well as higher gestational weight gain, early pregnancy FPG and FPG measurement gestational age (all *p* < 0.05). No significant differences existed in pre-pregnancy BMI, parity, past GDM/family diabetes history, macrosomic delivery history or pregnancy chronic hypertension (all *p* > 0.05).

In terms of pregnancy outcomes, the incidence rates of preeclampsia, premature rupture of membranes, cesarean section, preterm birth, postpartum hemorrhage, neonatal hyperbilirubinemia, neonatal asphyxia, LGA/Discordant LGA in twins and SGA/Discordant SGA in twins were significantly higher in the twin pregnancy group compared to the singleton pregnancy group (all *p* < 0.05). However, no statistically significant difference was found in the incidence of macrosomia between the two groups (*p* > 0.05). A detailed comparison of baseline characteristics and pregnancy outcomes between the singleton and twin pregnancy groups is presented in Table 2.

**Table 2:** Baseline characteristics of singleton and twin pregnancies.

Indicators	Singleton pregnancy *n* = 5,532	Twin pregnancy *n* = 175	*t*/*χ*^2^	*p*
Age			294.011	< 0.001
<35 years	5,042 (91.14)	90 (51.43)		
≥35 years	490 (8.86)	85 (48.57)		
Pre-pregnancy BMI			1.006	0.317
< 25 kg/m^2^	4,718 (85.29)	154 (88.00)		
≥ 25 kg/m^2^	814 (14.71)	21 (12.00)		
Weight Gain during Pregnancy, kg	12.41 ± 3.82	15.64 ± 4.26	10.972	< 0.001
Number of pregnancies			10.010	0.002
0 ~ 1 times	4,613 (83.39)	130 (74.29)		
≥ 2 times	919 (16.61)	45 (25.71)		
Order of birth			2.327	0.127
First birth	4,044 (73.10)	137 (78.29)		
Multiparity	1,488 (26.90)	38 (21.71)		
Assisted reproductive technology			675.223	< 0.001
Yes	271 (4.90)	94 (53.71)		
No	5,261 (95.10)	81 (46.29)		
Gestational age was measured by FPG in early pregnancy	6.84 ± 2.16	7.25 ± 2.14	2.473	0.013
FPG in early pregnancy, mmol/L	4.62 ± 0.39	4.73 ± 0.42	3.665	< 0.001
Gestational age at delivery	39 (38 ~ 40)	36 (35 ~ 37)	0.417	0.602
Pregnant women at high risk	2,214 (40.02)	115 (65.71)	46.357	< 0.001
History of GDM	298 (5.39)	10 (5.71)	0.036	0.850
Family history of diabetes	517 (9.35)	21 (12.00)	1.400	0.237
PCOS	184 (3.33)	14 (8.00)	11.065	0.001
History of macrosomia delivery	158 (2.86)	3 (1.71)	0.444	0.505
Chronic HTN in pregnancy	79 (1.43)	5 (2.86)	1.505	0.220
GDM	1,216 (21.98)	53 (30.29)	6.766	0.009
Preeclampsia	341 (6.16)	30 (17.14)	33.639	< 0.001
Premature rupture of membranes	1,007 (18.20)	52 (29.71)	14.873	< 0.001
Cesarean section	1,330 (24.04)	126 (72.00)	205.305	< 0.001
Preterm birth	219 (3.96)	93 (53.14)	794.336	< 0.001
Macrosomia	219 (3.96)	4 (2.29)	1.263	0.261
Postpartum Hemorrhage	315 (5.69)	31 (17.71)	43.035	< 0.001
Neonatal hyperbilirubinemia	455 (8.22)	40 (22.86)	45.850	< 0.001
Asphyxia neonatorum	96 (1.74)	9 (5.14)	9.101	0.002
LGA/Discordant LGA in twins	811 (14.66)	10 (5.71)	11.022	< 0.001
SGA/Discordant SGA in twins	1,510 (27.30)	17 (9.71)	26.756	< 0.001

Values are presented as number (%) or mean ± standard deviation.

FPG, fasting plasma glucose; BMI, body mass index; GDM, gestational diabetes mellitus; PCOS, polycystic ovary syndrome; LGA, large for gestational age; SGA, small for gestational age.

### Association of early pregnancy FPG and risk factors with GDM in singleton and twin pregnancies

Multivariable logistic regression indicated that among singleton gestations, both first-trimester FPG and every conventional risk factor—encompassing maternal age, pre-pregnancy BMI, history of GDM, family history of diabetes, PCOS, history of macrosomia delivery, and chronic HTN in pregnancy, demonstrated significant associations with incident GDM (all *p* < 0.05). For twin gestations, excluding history of GDM, and PCOS, first-trimester FPG and other remaining predictors maintained significant relationships with GDM development (all *p* < 0.05). Effect modification analyses revealed significant heterogeneity for pre-pregnancy BMI (*p*-interaction = 0.017) and PCOS (*p*-interaction = 0.033), with effect magnitudes markedly stronger in singleton versus twin gestations. Conversely, interaction *p*-values exceeded 0.05 for FPG, age, history of GDM, family history of diabetes, history of macrosomia delivery, and chronic HTN in pregnancy, indicating comparable predictive effects across pregnancy types (Table 3).

**Table 3:** Comparison of the association of FPG and risk factors with GDM between singleton and twin pregnancies.

Indicators	Singleton pregnancy			Twin pregnancy			*p*-interaction
	OR (95% CI)		*p*	OR (95% CI)		*p*	
FPG	1.829	1.719 ~ 1.956	0.010	2.103	1.751 ~ 2.963	0.010	0.483
Age	2.589	1.370 ~ 4.890	< 0.001	4.612	2.052 ~ 10.364	< 0.001	0.351
Pre-pregnancy BMI	2.804	1.655 ~ 4.751	< 0.001	4.389	1.467 ~ 13.131	0.008	0.017
History of GDM	5.193	2.407 ~ 11.206	< 0.001	3.611	0.646 ~ 20.183	0.144	0.528
Family history of diabetes	4.071	1.329 ~ 9.855	< 0.001	7.199	2.264 ~ 22.893	< 0.001	0.490
PCOS	3.619	1.329 ~ 9.855	0.001	2.578	0.638 ~ 10.410	0.184	0.033
History of macrosomia delivery	4.597	1.549 ~ 13.642	< 0.001	4.658	1.369 ~ 111.725	0.013	0.701
Chronic HTN in pregnancy	13.409	2.582 ~ 69.632	0.001	17.829	1.560 ~ 203.760	0.020	0.276

FPG, fasting plasma glucose; BMI, body mass index; GDM, gestational diabetes mellitus; PCOS, polycystic ovary syndrome.

**Figure 2:**
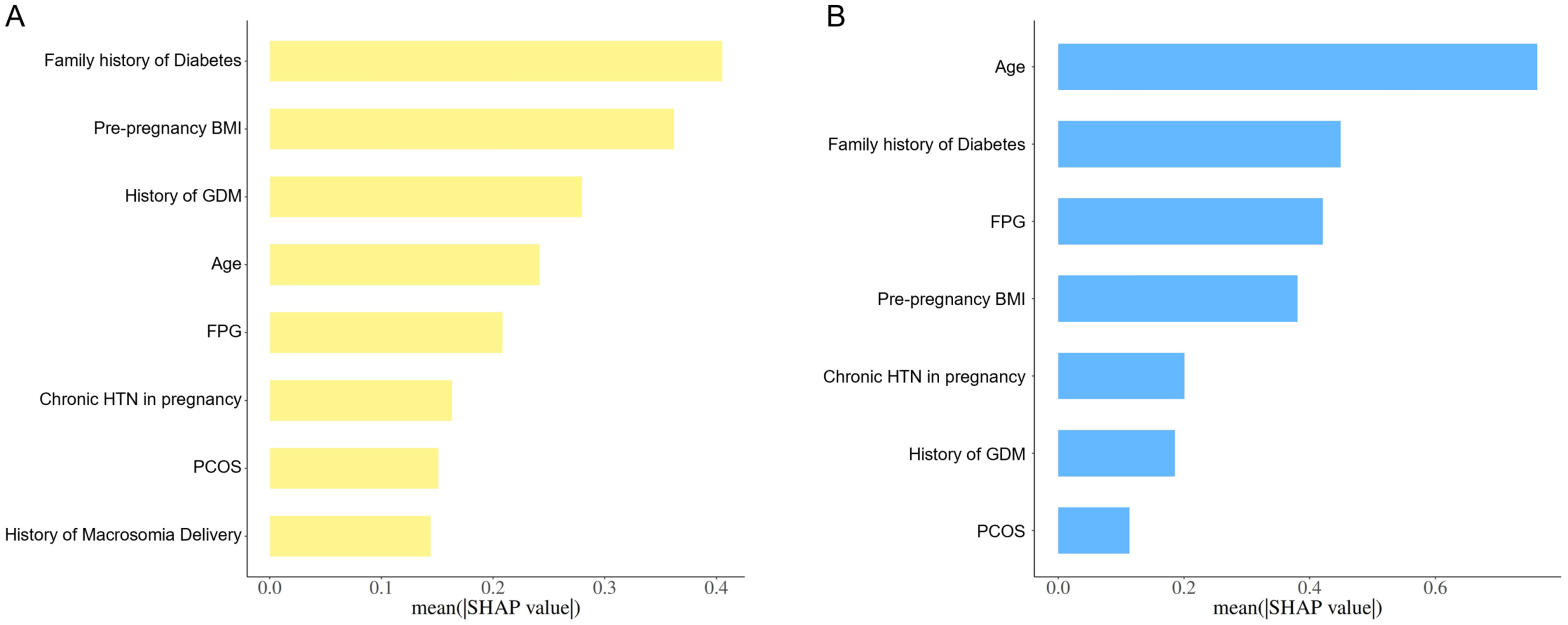
SHAP summary plot of factors associated with GDM in singleton and twin pregnancies: **(A)** Singleton pregnancy; **(B)** twin pregnancy.

The SHAP model was further employed to evaluate the contribution of each factor to GDM occurrence (Figure 2). The results showed that in singleton pregnancies, the top five factors contributing to GDM prediction were, in descending order: early pregnancy FPG, pre-pregnancy BMI, age, family history of diabetes, and history of GDM. In twin pregnancies, the top five contributing factors were: early pregnancy FPG, age, pre-pregnancy BMI, family history of diabetes, and history of macrosomia delivery. Of note, the contributions of history of GDM and PCOS were substantially lower in twin pregnancies compared to singleton pregnancies, consistent with the findings of the interaction analysis.

### Predictive performance of FPG integrated with clinical risk factors for GDM detection

**Figure 3:**
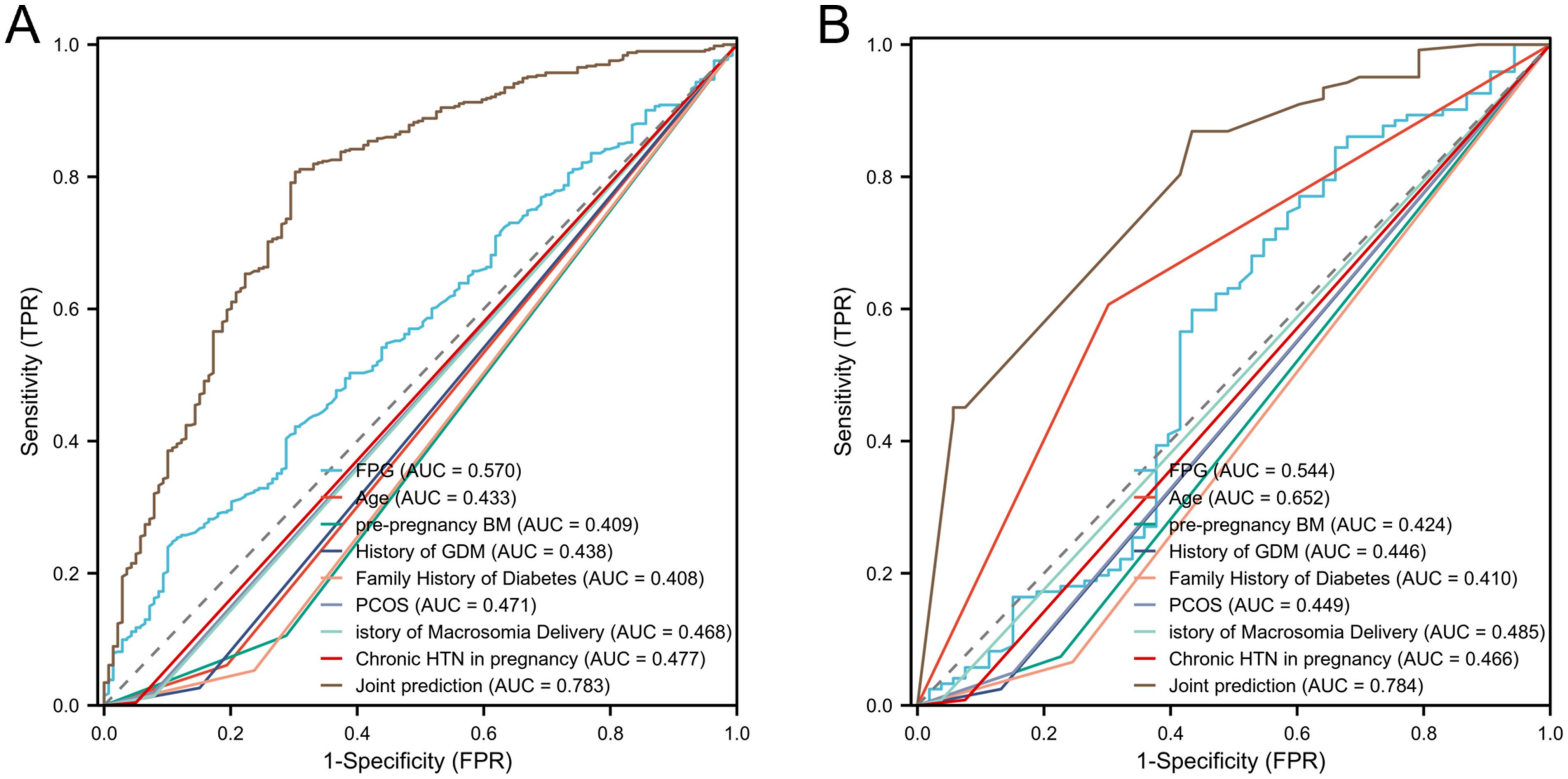
ROC curves of early pregnancy FPG and risk factors for predicting GDM in singleton and twin pregnancies: **(A)** Singleton pregnancy; **(B)** twin pregnancy.

ROC analysis indicated that isolated first-trimester FPG and single clinical predictors possessed modest discriminatory capacity for GDM across both gestation types, with no individual AUC exceeding 0.60. Specifically, FPG alone yielded AUCs of 0.570 among singletons and 0.544 among twins. Age demonstrated AUCs of 0.433 and 0.652, respectively. Remaining standalone predictors showed discrimination near chance level (AUC ≈ 0.50) regardless of fetal number. Conversely, incorporating FPG into a composite algorithm with all covariates markedly enhanced predictive accuracy, achieving AUCs of 0.783 and 0.784 for singleton and twin cohorts—both indicating satisfactory discrimination (Table 4; Figure 3).

**Table 4:** Predictive value of FPG and risk factors for GDM in singleton and twin pregnancies.

Predictor	Singleton pregnancy				
	Cut-off value	Sensitivity	Specificity	AUC	95% CI
FPG	4.885	0.247	0.892	0.570	0.518 ~ 0.622
Age	30.570	0.596	0.841	0.433	0.399 ~ 0.468
Pre-pregnancy BMI	26.730	0.615	0.708	0.409	0.369 ~ 0.449
History of GDM	-	0.151	0.026	0.438	0.407 ~ 0.468
Family history of diabetes	-	0.237	0.053	0.408	0.371 ~ 0.445
PCOS	-	0.079	0.020	0.471	0.447 ~ 0.494
History of macrosomia delivery	-	0.079	0.014	0.468	0.444 ~ 0.491
Chronic HTN in pregnancy	-	0.050	0.004	0.477	0.458 ~ 0.495
Joint prediction	0.207	0.807	0.698	0.783	0.738 ~ 0.828

Predictor	Twin pregnancy				
	Cut-off value	Sensitivity	Specificity	AUC	95% CI
FPG	5.260	0.844	0.340	0.544	0.442 ~ 0.647
Age	28.31	0.607	0.698	0.652	0.576 ~ 0.728
Pre-pregnancy BMI	24.82	0.563	0.672	0.424	0.362 ~ 0.485
History of GDM	-	0.151	0.026	0.446	0.398 ~ 0.494
Family history of diabetes	-	0.237	0.053	0.410	0.348 ~ 0.473
PCOS	-	0.079	0.020	0.449	0.397 ~ 0.501
History of macrosomia delivery	-	0.079	0.014	0.485	0.458 ~ 0.512
Chronic HTN in pregnancy	-	0.050	0.004	0.466	0.430 ~ 0.503
Joint prediction	0.370	0.869	0.566	0.784	0.715 ~ 0.853

### Association of early pregnancy FPG and risk factors with adverse perinatal outcomes

Multivariable logistic regression—conducted with and without covariate adjustment—demonstrated that baseline FPG alongside clinical risk factors maintained significant relationships with preeclampsia, premature membrane rupture, operative delivery, spontaneous preterm birth, postpartum hemorrhage, fetal macrosomia, neonatal jaundice, birth asphyxia, LGA/Discordant LGA (twins), and SGA/Discordant SGA (twins) among singleton gestations (all *p* < 0.05). Notably, the preeclampsia association attenuated to non-significance following adjustment (*p* = 0.744). Among twin gestations, FPG and accompanying risk factors correlated significantly with every aforementioned complication both pre- and post-adjustment (all *p* < 0.05). Moreover, effect magnitudes—quantified by adjusted odds ratios—were markedly elevated in twins versus singletons, suggesting amplified vulnerability of multiple gestations to FPG-related metabolic perturbations.

Interaction analysis identified significant heterogeneity for preeclampsia, membrane rupture, cesarean delivery, preterm birth, hemorrhage, and macrosomia across pregnancy types (*p*-interaction < 0.05). Conversely, neonatal jaundice and asphyxia showed comparable effect profiles (*p*-interaction > 0.6). Detailed data are presented in Table 5.

**Table 5:** Association of early pregnancy FPG with adverse perinatal outcomes in singleton and twin pregnancies.

Perinatal outcomes	Singleton pregnancy				*p*-interaction
	OR (95%CI)	*p*	Adjusted OR (95% CI)	*p*	
Preeclampsia	1.387 (1.118 ~ 1.720)	0.003	0.964 (0.776 ~ 1.198)	0.744	< 0.001
Premature rupture of membranes	1.238 (1.083 ~ 1.416)	0.002	1.264 (1.101 ~ 1.452)	< 0.001	< 0.001
Cesarean section	1.170 (1.037 ~ 1.319)	0.011	1.194 (1.053 ~ 1.353)	0.006	< 0.001
Preterm birth	1.94 (1.46 ~ 2.57)	< 0.001	1.981 (1.480 ~ 2.652)	< 0.001	< 0.001
Macrosomia	1.260 (1.011 ~ 1.571)	0.004	1.260 (1.011 ~ 1.571)	0.040	< 0.001
Postpartum Hemorrhage	1.388 (1.066 ~ 1.807)	0.015	1.436 (1.086 ~ 1.897)	0.011	0.048
Neonatal hyperbilirubinemia	2.06 (1.653 ~ 2.567)	< 0.001	2.056 (1.649 ~ 2.564)	< 0.001	0.630
Asphyxia neonatorum	2.31 (1.510 ~ 3.535)	< 0.001	2.307 (1.508 ~ 3.529)	< 0.001	0.712
LGA/Discordant LGA in twins	1.215 (1.114 ~ 1.596)	0.018	1.219 (1.117 ~ 1.607)	0.024	0.419
SGA/Discordant SGA in twins	1.136 (1.058 ~ 1.269)	0.009	1.130 (1.052 ~ 1.251)	0.014	0.883

Perinatal outcomes	Twin pregnancy				*p*-interaction
	OR (95% CI)	*p*	Adjusted OR (95% CI)	*p*	
Preeclampsia	3.197 (2.053 ~ 4.979)	< 0.001	3.112 (2.002 ~ 4.837)	< 0.001	< 0.001
Premature rupture of membranes	6.275 (3.532 ~ 11.150)	< 0.001	6.301 (3.546 ~ 11.194)	< 0.001	< 0.001
Cesarean section	3.232 (2.179 ~ 4.795)	< 0.001	3.232 (2.179 ~ 4.795)	< 0.001	< 0.001
Preterm birth	4.916 (3.038 ~ 7.955)	< 0.001	5.915 (3.441 ~ 10.168)	< 0.001	< 0.001
Macrosomia	3.543 (2.215 ~ 5.669)	< 0.001	3.543 (2.215 ~ 5.669)	< 0.001	< 0.001
Postpartum hemorrhage	3.270 (1.623 ~ 6.587)	< 0.001	3.270 (1.623 ~ 6.587)	< 0.001	0.048
Neonatal hyperbilirubinemia	2.235 (1.620 ~ 3.084)	< 0.001	2.235 (1.620 ~ 3.084)	< 0.001	0.630
Asphyxia neonatorum	2.798 (1.645 ~ 4.758)	< 0.001	2.798 (1.645 ~ 4.758)	< 0.001	0.712
LGA/Discordant LGA in twins	2.264 (1.532 ~ 3.158)	< 0.001	2.283 (1.572 ~ 3.206)	< 0.001	0.419
SGA/Discordant SGA in twins	1.725 (1.526 ~ 3.034)	< 0.001	1.736 (1.529 ~ 3.038)	< 0.001	0.883

## Discussion

This study compared clinical characteristics between singleton and twin pregnancies and examined how early pregnancy FPG and traditional risk factors differentially affect GDM risk and perinatal outcomes. Twin pregnancy was confirmed as a high-risk condition for GDM and adverse outcomes. While early pregnancy FPG and most traditional risk factors predicted GDM in both groups, pre-pregnancy BMI and PCOS showed distinct effects. These findings support risk-stratified, individualized management in clinical practice.

Our results showed that the incidence of GDM was significantly higher in twin pregnancies than in singleton pregnancies (30.29% vs. 21.98%), consistent with previous reports (14). Of note, the GDM incidence observed in both pregnancy groups in our study was higher than that reported in Western populations, a finding that aligns with well-documented ethnic disparities in GDM prevalence. A recent systematic review and meta-analysis incorporating 1,105 Chinese studies (1990–2024) reported a pooled GDM prevalence of 15.6% (95% CI: 14.9–16.2%) based on the IADPSG criteria, which is 2–3 times higher than rates obtained using other diagnostic criteria (15). Globally, the pooled GDM prevalence in Asian populations reaches 20.9%, significantly exceeding that in other ethnic groups (16). The higher incidence observed in our cohort may be attributed to the following three factors: (1) strict application of the IADPSG one-step diagnostic criteria; (2) increasing trends in maternal age and obesity in China; and (3) genetic predisposition in East Asian populations characterized by higher insulin resistance at lower BMI thresholds (17). These ethnicity-specific factors suggest that our findings should be extrapolated to non-Asian populations with caution and warrant validation studies in diverse ethnic cohorts. Having explained the ethnic background underlying the overall higher incidence, we further analyzed the specific mechanisms contributing to the elevated GDM risk in twin pregnancies. Most studies attribute the difference between twin and singleton pregnancies to the higher baseline risk profile of twin gestations, and our findings further support this view: the proportions of advanced maternal age (≥35 years) and assisted reproductive technology conception were significantly higher in the twin pregnancy group, both of which promote the progression of insulin resistance by impairing pancreatic *β*-cell function and exacerbating chronic low-grade inflammation and endocrine disorders (18, 19). Unlike singleton pregnancies, twin pregnancies are characterized by enlarged placental volume and markedly increased secretion of placenta-derived insulin-antagonistic hormones, which, combined with doubled maternal nutritional demands and metabolic load, collectively create a stronger physiological insulin resistance environment (20). Furthermore, we observed higher proportions of polycystic ovary syndrome and high-risk pregnancy designation in twin pregnancies, suggesting that the synergistic effect of multiple insulin resistance-related risk factors collectively elevates the risk of GDM (21). This finding aligns with the conclusion of previous mechanistic studies that “the interplay of multiple risk factors amplifies metabolic abnormalities” (22).

Regarding perinatal morbidity, this investigation demonstrated significantly augmented frequencies of hypertensive disorders, spontaneous membrane rupture, iatrogenic and spontaneous preterm delivery, operative birth, excessive bleeding, and neonatal pathology among multiple gestations. These observations converge with contemporary large-scale retrospective analyses (23, 24). Underlying mechanisms stem from distinctive anatomical-physiological adaptations in twin conceptions: excessive uterine distension elevates membrane rupture and preterm labor risks; pronounced circulating volume expansion coupled with hemodynamic volatility predisposes to endothelial injury, precipitating hypertensive disease (19, 20); the clustering of obstetric complications and considerations for delivery safety collectively contribute to the elevated cesarean section rate; and relative placental insufficiency, intrauterine nutritional competition, iatrogenic preterm birth, and intrauterine hypoxia are the core drivers of adverse neonatal outcomes (25, 26). Importantly, there was no statistically significant difference in the incidence of macrosomia between the two groups. This finding aligns with the conclusions of some single-center studies but diverges from certain studies based on Western populations (27, 28). We propose that factors such as limited intrauterine space, uneven placental blood supply distribution, and inter-fetal nutritional competition in twin pregnancies may, to some extent, restrict excessive fetal growth, thereby counteracting the risk of macrosomia induced by hyperglycemia. This suggests that the mechanism of macrosomia in twin pregnancies is more constrained by the placental-fetal unit rather than being solely determined by maternal glucose metabolism. Furthermore, this study demonstrated that the incidences of LGA, discordant LGA in twins, SGA, and discordant SGA in twins were significantly higher in twin pregnancies. The primary reasons for these findings are closely related to placental sharing, uneven blood flow distribution, intrauterine growth competition, and relative placental functional insufficiency in twin gestations (29, 30). These findings further suggest that abnormal fetal growth in twin pregnancies is characterized by growth discordance. Therefore, clinical monitoring should prioritize the assessment of inter-twin birth weight differences, providing a reference for optimizing fetal weight management strategies in twin pregnancies.

In this study, ROC curve analysis revealed that individual predictors, including early pregnancy FPG, had limited value for GDM prediction, with all AUCs falling below 0.6 in both singleton and twin pregnancies. This aligns with recent evidence: Bhattacharya et al. (31) reported a pooled AUC of approximately 0.63 (95% CI: 0.59–0.67) for isolated first-trimester FPG in a 2024 meta-analysis of 47 studies, with substantial heterogeneity (I^2^ = 85%). Gao et al. (32) further demonstrated that the predictive value of early pregnancy FPG for adverse outcomes in Chinese women varies by gestational age (AUCs 0.65–0.72). In stark contrast, the combined model, integrating early pregnancy FPG with all risk factors, exhibited a substantial improvement in predictive performance, achieving AUCs of 0.783 and 0.784 for singleton and twin pregnancies, respectively. This performance is comparable to recent international reports: Sontag et al. (33) achieved an AUC of 0.83 using cluster analysis of the German GestDiab cohort, while Cidade-Rodrigues et al. (34) reported moderate predictive value (AUC ≈ 0.65) for first-trimester FPG ≥ 5.1 mmol/L alone. This finding underscores the multifactorial nature of GDM pathophysiology (35, 36). The superior performance of the combined model suggests that it can more comprehensively capture the complex interplay between baseline metabolic status (reflected by FPG) and individual risk profiles (37). Consequently, this approach holds strong potential for clinical implementation, enabling the early stratification of high-risk gravidas in both pregnancy types and paving the way for timely, targeted interventions prior to the standard 24–28 week diagnostic window.

Multivariate regression and interaction analyses revealed differences in the pathogenesis of GDM between singleton and twin pregnancies. In singleton pregnancies, early pregnancy FPG and all traditional risk factors were significantly associated with GDM, consistent with the results of previous studies (38). In contrast, in twin pregnancies, the associations of GDM history and PCOS with GDM were not statistically significant, and the effects of pre-pregnancy BMI and PCOS on GDM were significantly weaker than those observed in singleton pregnancies. SHAP analysis further confirmed the attenuated predictive roles of GDM history and PCOS in twin pregnancies. The underlying reason is that the high metabolic load and pronounced insulin resistance induced by twin gestation become dominant drivers of GDM occurrence, thereby masking the independent effects of these baseline risk factors (39). Furthermore, this study found that early pregnancy FPG and risk factors were significantly associated with adverse perinatal outcomes, with more pronounced effects observed in twin pregnancies that persisted after adjusting for confounders. These results are consistent with the findings of Batsry L et al. (40). We speculate that this may be attributed to the impact of early pregnancy glucose metabolic abnormalities on fetal and placental development, combined with the synergistic effects of risk factors and the heightened metabolic stress characteristic of twin gestations (41). Therefore, for twin pregnancies, particularly those with high-risk factors, intensified glycemic management and comprehensive pregnancy monitoring are essential to improve maternal and neonatal outcomes.

This study also revealed that early pregnancy FPG and associated risk factors are linked not only to GDM but also to a range of adverse perinatal outcomes, with particularly notable effects observed in twin pregnancies. Even after controlling for confounding variables, early pregnancy FPG and risk factors continued to show a significant association with all adverse outcomes in twin pregnancies, exhibiting larger effect sizes. This indicates a more substantial and lasting negative impact on both mothers and newborns in these cases. This phenomenon may be attributed to the fact that early pregnancy is a crucial phase for fetal organ development and placental formation; any abnormalities in glucose metabolism can lead to fetal hyperinsulinemia, adversely affecting development (42). Additionally, the presence of risk factors, when combined with gestational stress, further increases the likelihood of adverse outcomes (43). Consequently, for twin pregnancies, especially those with identifiable risk factors, it is essential to enhance comprehensive pregnancy monitoring—such as preeclampsia prevention and assessments of fetal intrauterine status—alongside effective glycemic management to improve outcomes for both mothers and their infants.

## Limitations

Several limitations should be acknowledged. First, a notable limitation of this study is the substantial disparity in sample sizes between the singleton (*n* = 5,532) and twin (*n* = 175) cohorts. While this reflects the genuine epidemiological distribution of pregnancy types, the smaller twin cohort may have reduced statistical power, increasing the risk of Type II errors. Consequently, the non-significant findings for certain risk factors in the twin group should be interpreted with caution. Second, the single-center retrospective nature of this study, with all data derived from Ningjin County Hospital in Hebei Province, may introduce selection bias and thus constrain the broader applicability of our findings. Third, the failure to include placental function indicators as mediating or moderating variables in the analysis of the relationship between first-trimester FPG and GDM restricts a deeper understanding of the mechanisms by which early pregnancy glucose metabolism contributes to GDM. Fourth, the study population was exclusively of East Asian ancestry, which limits generalizability to other ethnic groups with different underlying pathophysiological mechanisms and GDM prevalence patterns. Future multicenter prospective cohort studies are warranted to further validate and refine early pregnancy combined screening strategies, explore the mediating roles of placental function indicators, and establish a comprehensive life-course management system for GDM through extended follow-up.

## Conclusion

In summary, multiple gestation carries markedly elevated risks for both GDM onset and perinatal morbidity relative to singleton conception. First-trimester FPG, together with the majority of conventional predisposing factors, serves as a shared predictor of GDM across pregnancy types; however, pre-gravid BMI and PCOS demonstrate differentially stronger effects in singletons. Integrating FPG with clinical risk profiles yields satisfactory screening performance regardless of fetal number. Clinically, priority should be assigned to first-trimester combined screening protocols, with management strategies tailored to pregnancy-specific risk factor profiles. Heightened surveillance is particularly warranted for twin gestations, advanced maternal age, and assisted conception, aiming to curtail GDM incidence and perinatal complications while optimizing maternal-neonatal outcomes.

## Data Availability

The raw data supporting the conclusions of this article will be made available by the authors, without undue reservation.

## References

[1] Goyal A Gupta Y Tandon N. Overt diabetes in pregnancy. Diabetes Ther. (2022) 13:589–600. doi: 10.1007/s13300-022-01210-6, 35107789 PMC8991291

[2] Greenberg M Zhu Y Shan J Hedderson MM Ngo A Quesenberry CP . Gestational and Pregestational diabetes screening changes in early pregnancy and perinatal outcomes. Obstet Gynecol. (2026) 147:209–20. doi: 10.1097/AOG.0000000000006090, 41100860

[3] Bastian B Smithers LG Pape A Davis W Fu K Francois M. Early screening and diagnosis of gestational diabetes mellitus (GDM) and its impact on perinatal outcomes. Diabetes Res Clin Pract. (2024) 217:111890. doi: 10.1016/j.diabres.2024.111890, 39433214

[4] Liu T Gao R Liu Y Zhao K Su X Wong HC . Hypertensive disorders of pregnancy and neonatal outcomes in twin vs. singleton pregnancies after assisted reproductive technology. Front Pediatr. (2022) 10:839882. doi: 10.3389/fped.2022.839882, 36120650 PMC9478585

[5] Lin D Huang X Fan D Chen G Li P Rao J . Factors and perinatal outcomes associated with intrapartum cesarean delivery in twin gestations. Obstet Gynecol. (2025) 146:245–54. doi: 10.1097/AOG.0000000000005912, 40373309

[6] Wang L Qin M Chen GQ Wang Y Zhang D Chen Q . Abnormal fasting glucose levels in the diagnosis of GDM may be associated with adverse pregnancy outcomes. Arch Gynecol Obstet. (2026) 313:44. doi: 10.1007/s00404-025-08266-2, 41538074 PMC12808146

[7] Hanson E Ringmets I Kirss A Laan M Rull K. Screening of gestational diabetes and its risk factors: pregnancy outcome of women with gestational diabetes risk factors according to glycose tolerance test results. J Clin Med. (2022) 11:4953. doi: 10.3390/jcm11174953, 36078883 PMC9456276

[8] Lautredou M Pan-Petesch B Dupré PF Drugmanne G Nowak E Anouilh F . Excessive gestational weight gain is an independent risk factor for gestational diabetes mellitus in singleton pregnancies: results from a French cohort study. Eur J Obstet Gynecol Reprod Biol. (2022) 275:31–6. doi: 10.1016/j.ejogrb.2022.06.009, 35714502

[9] International Association of Diabetes and Pregnancy Study Groups Consensus Panel Metzger BE Gabbe SG Persson B Buchanan TA Catalano PA . International association of diabetes and pregnancy study groups recommendations on the diagnosis and classification of hyperglycemia in pregnancy. Diabetes Care. (2010) 33:676–82. doi: 10.2337/dc09-1848 20190296 PMC2827530

[10] Sweeting A Wong J Murphy HR Ross GP. A clinical update on gestational diabetes mellitus. Endocr Rev. (2022) 43:763–93. doi: 10.1210/endrev/bnac003, 35041752 PMC9512153

[11] Li P Lin S Li L Cui J Zhou S Fan J. First-trimester fasting plasma glucose as a predictor of gestational diabetes mellitus and the association with adverse pregnancy outcomes. Pak J Med Sci. (2019) 35:95–100. doi: 10.12669/pjms.35.1.216, 30881404 PMC6408635

[12] Wang C Juan J Yang H. A summary of Chinese guidelines on diagnosis and Management of Hyperglycemia in pregnancy (2022). Matern Fetal Med. (2023) 5:4–8. doi: 10.1097/FM9.0000000000000181, 40433235 PMC12106219

[13] Dai L Deng C Li Y Yi L Li X Mu Y . Population-based birth weight reference percentiles for Chinese twins. Ann Med. (2017) 49:470–8. doi: 10.1080/07853890.2017.1294258, 28276868

[14] Ashwal E Berger H Hiersch L Yoon EW Zaltz A Shah B . Gestational diabetes and fetal growth in twin compared with singleton pregnancies. Am J Obstet Gynecol. (2021) 225:420.e1–420.e13. doi: 10.1016/j.ajog.2021.04.225, 33872592

[15] Wu X Tiemeier H Xu T. Trends in gestational diabetes prevalence in China from 1990 to 2024: a systematic review and meta-analysis. Rev Endocrine Metabolic Disord. (2025) 26:1009–21. doi: 10.1007/s11154-025-09987-0, 40663307

[16] Lee KW Ching SM Ramachandran V Yee A Hoo FK Chia YC . Prevalence and risk factors of gestational diabetes mellitus in Asia: a systematic review and meta-analysis. BMC Pregnancy Childbirth. (2018) 18:494. doi: 10.1186/s12884-018-2131-4, 30547769 PMC6295048

[17] Moon JH Kwak SH Jang HC. Prevention of type 2 diabetes mellitus in women with previous gestational diabetes mellitus. Korean J Intern Med. (2017) 32:26–41. doi: 10.3904/kjim.2016.203, 28049284 PMC5214732

[18] Vitagliano A Paffoni A Viganò P. Does maternal age affect assisted reproduction technology success rates after euploid embryo transfer? A systematic review and meta-analysis. Fertil Steril. (2023) 120:251–65. doi: 10.1016/j.fertnstert.2023.02.036, 36878347

[19] Lou Y He P Jiang H Xiang L Gao X. Analysis of the characteristics of blood lipid metabolism in twin pregnancy. J Investig Med. (2023) 71:53–7. doi: 10.1136/jim-2022-002412, 36137709

[20] Melamed N Avnon T Barrett J Fox N Rebarber A Shah BR . Gestational diabetes in twin pregnancies-a pathology requiring treatment or a benign physiological adaptation? Am J Obstet Gynecol. (2024) 231:92–104.e4. doi: 10.1016/j.ajog.2024.01.004, 38218511

[21] Løvvik TS Wikström AK Neovius M Stephansson O Roos N Vanky E. Pregnancy and perinatal outcomes in women with polycystic ovary syndrome and twin births: a population-based cohort study. BJOG. (2015) 122:1295–302. doi: 10.1111/1471-0528.13339, 25761516

[22] Guillén-Sacoto MA Barquiel B Hillman N Burgos MA Herranz L. Metabolic syndrome and impaired glucose metabolism during early postpartum after twin pregnancies complicated by gestational diabetes mellitus: is the risk comparable to singleton pregnancies? Diabetes Metab. (2019) 45:390–3. doi: 10.1016/j.diabet.2017.10.008, 29169927

[23] Che R Pei J Chen H Dong L Wu Y Hua X. The influence of hypertensive disorders during pregnancy on the perinatal outcome of different chorionic twins. J Matern Fetal Neonatal Med. (2022) 35:7146–52. doi: 10.1080/14767058.2021.1945574, 34180344

[24] Liu L Yang HS Xu Z Meng L Lu Y Han L . Explore the impact of abnormal coagulation test results on pregnancy complications and perinatal outcomes by establishing the trimester-specific reference intervals of singleton and twin pregnancies. Clin Chim Acta. (2023) 541:117265. doi: 10.1016/j.cca.2023.117265, 36801269

[25] Larsen NL Koefoed AS Kampmann U Retnakaran R Ovesen PG Fuglsang J. Gestational diabetes mellitus in twin pregnancies versus singleton pregnancies-a retrospective case-control study. Diabet Med. (2025) 42:e70095. doi: 10.1111/dme.70095, 40548386 PMC12434430

[26] Kotaki H Tachihara M Kamiya M Shimabukuro M Sakuma J Takano M . Maternal cardiovascular dynamics in twin pregnancies complicated by twin-to-twin transfusion syndrome. J Obstet Gynaecol Res. (2025) 51:e16224. doi: 10.1111/jog.16224, 39891372

[27] Berezowsky A Ardestani S Hiersch L Shah BR Berger H Halperin I . Glycemic control and neonatal outcomes in twin pregnancies with gestational diabetes mellitus. Am J Obstet Gynecol. (2023) 229:682.e1–682.e13. doi: 10.1016/j.ajog.2023.06.046, 37393013

[28] Guillén-Sacoto MA Barquiel B Hillman N Burgos MÁ Herranz L. Gestational diabetes mellitus: glycemic control during pregnancy and neonatal outcomes of twin and singleton pregnancies. Endocrinología Diabetes y Nutrición. (2018) 65:319–27. doi: 10.1016/j.endinu.2018.01.011, 29685731

[29] Luo J Huang W Luo S Deng L Lin L Liao Q . Assessing the risk factors and establishing multivariable prediction models for singleton macrosomia. Front Med. (2025) 12:1590283. doi: 10.3389/fmed.2025.1590283, 41070070 PMC12504509

[30] Guillén MA Herranz L Barquiel B Hillman N Burgos MA Pallardo LF. Influence of gestational diabetes mellitus on neonatal weight outcome in twin pregnancies. Diabet Med. (2014) 31:1651–6. doi: 10.1111/dme.12523, 24925592

[31] Bhattacharya S Nagendra L Dutta D Mondal S Bhat S Raj JM . First-trimester fasting plasma glucose as a predictor of subsequent gestational diabetes mellitus and adverse fetomaternal outcomes: a systematic review and meta-analysis. Diabetes Metab Syndr Clin Res Rev. (2024) 18:103051. doi: 10.1016/j.dsx.2024.103051, 38843646

[32] Gao M Wang H Li N Li W Zhang T Qiao Y . High fasting plasma glucose in early pregnancy and postpartum diabetes in Chinese women with gestational diabetes. Diabetes Care. (2026) 49:e49–51. doi: 10.2337/dc25-2310, 41529158

[33] Sontag I Kschischo M Kaltheuner M Jander L Leubner P Adamczewski H . Novel definition of time range and risk factors of pregnant women with gestational diabetes mellitus detected early in pregnancy a cluster analysis using clinical data of the German GestDiab cohort. Diabetol Metab Syndr. (2025) 17:426. doi: 10.1186/s13098-025-02000-3, 41233894 PMC12616929

[34] Cidade-Rodrigues C Silva B Silva VB Chaves C Mazeda ML Araújo A . Untreated women with first trimester fasting glycaemia 92−125 mg/dL and risk of gestational diabetes mellitus in the 24−28th week OGTT: prevalence and predictors. Acta Diabetol. (2025) 62:1291–8. doi: 10.1007/s00592-025-02450-1, 39821306

[35] Huang S Li X Zheng J Qiu G Luo P Mao L . Risk factors and pregnancy outcomes of twin pregnancies with gestational diabetes mellitus: a comparison based on Chorionicity. J Diabetes Res. (2026) 2026:9744892. doi: 10.1155/jdr/9744892, 41541003 PMC12800392

[36] Balke S Kotzott IA Aigner A Weid P Henrich W Dudenhausen JW . Gestational diabetes mellitus in singleton and twin pregnancies: a comparison of Fetomaternal outcomes. Diagnostics. (2026) 16:632. doi: 10.3390/diagnostics16040632, 41750780 PMC12939607

[37] Zavalza-Gómez AB Pérez-Hernández SC Arrazola-Fregozo AG Espinel-Bermúdez MC Grover-Páez F González SP . Percentage of change and increment of the glucose level after a normal oral tolerance curve between 24 and 28 weeks of pregnancy. J Obstet Gynaecol Res. (2023) 49:846–51. doi: 10.1111/jog.15517, 36482822

[38] Zheng Y Hou W Xiao J Huang H Quan W Chen Y. Application value of predictive model based on maternal coagulation function and glycolipid metabolism indicators in early diagnosis of gestational diabetes mellitus. Front Public Health. (2022) 10:850191. doi: 10.3389/fpubh.2022.850191, 35387184 PMC8978602

[39] Akiba Y Miyakoshi K Ikenoue S Saisho Y Kasuga Y Ochiai D . Glycemic and metabolic features in gestational diabetes: singleton versus twin pregnancies. Endocr J. (2019) 66:647–51. doi: 10.1507/endocrj.EJ18-0575, 31019153

[40] Batsry L Zloto K Kalter A Baum M Mazaki-Tovi S Yinon Y. Perinatal outcomes of intrahepatic cholestasis of pregnancy in twin versus singleton pregnancies: is plurality associated with adverse outcomes? Arch Gynecol Obstet. (2019) 300:881–7. doi: 10.1007/s00404-019-05247-0, 31346701

[41] Das D Christie HE Hegazi M Takawy M Pone KA Vella A . Twin pregnancy complicated by gestational diabetes mellitus: maternal and neonatal outcomes. J Endocr Soc. (2024) 8:bvae075. doi: 10.1210/jendso/bvae075, 38698871 PMC11065348

[42] Sesmilo G Prats P Garcia S Rodríguez I Rodríguez-Melcón A Berges I . First-trimester fasting glycemia as a predictor of gestational diabetes (GDM) and adverse pregnancy outcomes. Acta Diabetol. (2020) 57:697–703. doi: 10.1007/s00592-019-01474-8, 31984438

[43] Greco E Calanducci M Nicolaides KH Barry EVH Huda MSB Iliodromiti S. Gestational diabetes mellitus and adverse maternal and perinatal outcomes in twin and singleton pregnancies: a systematic review and meta-analysis. Am J Obstet Gynecol. (2024) 230:213–25. doi: 10.1016/j.ajog.2023.08.011, 37595821

